# Transcriptomic Analysis Divulges Differential Expressions of Microglial Genes After Microglial Repopulation in Mice

**DOI:** 10.3390/ijms26041494

**Published:** 2025-02-11

**Authors:** Muhammad Tariq Hafeez, Hao Gao, Furong Ju, Fujian Qi, Ting Li, Shengxiang Zhang

**Affiliations:** Gansu Key Laboratory of Biomonitoring and Bioremediation for Environmental Pollution, School of Life Sciences, Lanzhou University, Lanzhou 730000, China

**Keywords:** microglia, depletion, density, proliferation, RNA sequencing (RNA-Seq)

## Abstract

Microglia are key immune cells in the central nervous system (CNS) and maintain hemostasis in physiological conditions. Microglial depletion leads to rapid repopulation, but the gene expression and signaling pathways related to repopulation remain unclear. Here, we used RNA sequencing (RNA-Seq) analysis to profile the transcriptome of microglia-depleted tissue by taking advantage of a conditional genetic microglial depletion model (CX3CR1CreER/+ system). Differential gene expression (DGE) sequencing analysis showed that 1226 genes were differentially up- and downregulated in both groups compared to control. Our data demonstrated that many microglial genes were highly regulated on day 3 after depletion but the numbers of differentially expressed genes were reduced by day 7. Gene ontology (GO) analysis categorized these differentially expressed genes on day 3 and day 7 to the specific biological processes, such as cell proliferation, cell activation, and cytokine and chemokine production. DGE analysis indicated that specific genes related to proliferation were regulated after depletion. Consistent with the changes in transcriptome, the histological analysis of transgenic mice revealed that the microglia after depletion undergo proliferation and activation from day 3 to day 7. Collectively, these results suggest that transcriptomic changes in microglial genes during depletion have a profound implication for the renewal and activation of microglia and may help to understand the regulatory mechanism of microglial activation in disease conditions.

## 1. Introduction

Microglia are resident immune cells in the CNS that make up about 5–15% of the total number of cells in the CNS [1,2]. They arise from progenitor cells in the yolk sac during the embryonic period and are a special type of mononuclear macrophage [3,4,5]. Under physiological conditions, microglia are in a homeostatic state characterized by small cell diameters and branched morphologies, with many tiny protrusions extending hierarchically from the cell body. While the cell body does not move much, the protrusions of microglia are highly dynamic. They are constantly expanding, moving, and surveying the CNS from time to time [6].

Microglia are essential elements of the CNS and perform crucial homeostatic functions. As the first line of defense, microglia can directly or indirectly contact neurons, astrocytes, or oligodendrocytes via their multilevel branches and play a dynamic surveillance role in the CNS [7,8]. On the other hand, microglia function as a special type of secretory cells that can secrete trophic factors in the “homeostatic state” to regulate the activity of neurons and oligodendrocytes and thus influence important processes such as axon growth and myelination of neurons [9]. Therefore, microglia play a key regulatory role in the CNS under physiological conditions and at the same time represent an important immune surveillance system for the maintenance of CNS development and homeostasis.

Numerous data indicate that microglia are not only important for normal brain function but also play a crucial role in neurological diseases [10,11]. In particular, they can rapidly respond to potential pathological damage and quickly and effectively remove invading pathogens and cellular debris by phagocytosis to maintain the stability of the CNS neuronal network [12,13,14]. The absence or dysregulation of microglia can directly lead to abnormal immune regulation, inflammatory storms, and neuronal death in the CNS [15,16]. The acquisition of hypertrophic morphology, which is associated with microglial reactivity, is strongly correlated with the transcription of genes linked to cellular migration and phagocytosis [17], neuropathologies, including Alzheimer’s disease [18], Down syndrome [19], hippocampal sclerosis [20], LPS-induced neuroinflammation [21], neuraminidase-induced neuroinflammation [22], and neurotrauma [23]. These findings highlight the importance of understanding the molecular mechanisms underlying microglial reactivity and their implications in different neurological disorders.

Microglia can repopulate rapidly in response to pathological insults [24,25,26,27]. Some studies suggest that repopulating microglia are from infiltrating blood cells into the brain [28,29]. Other research indicates that the repopulation of microglia occurs from nestin-expressing cells throughout the CNS, which represent a microglial progenitor cell [4]. Additionally, it has been proposed that the repopulating microglia are derived from surviving microglia after depletion (<0.90%) [30]. Studies indicate that microglia can also rapidly repopulate in the microglia depletion model using acute diphtheria toxin and Pexidartinib (PLX3397) [31,32,33,34,35]. These studies confirm that the repopulation of microglia occurred within the CNS. As microglia maintain homeostasis, they also play a key role in the development and maintenance of the inflammatory response, showing enhanced proliferation and activation after depletion. However, the significant gene expression and signaling pathways associated with the proliferation and activation of microglia following depletion in the brain remain elusive.

In this study, through differential gene expression (DGE) technology, we aimed to develop a set of transcriptomic data from the brain tissue after microglial depletion using CX3CR1^CreER^: R26^iDTR^ mice. As a result, we found that the microglial genes were differentially expressed in the brain after microglial depletion and were also involved in the proliferation, activation, and chemokines of several signaling pathways. Data also revealed that numerous microglial genes were highly regulated in response to microglial depletion. Some key Gene Ontology (GO) terms and Kyoto Encyclopedia of the Genes and the Genomes (KEGG) pathways were extracted, such as the CSF1R, PTPRC, and PTGS1 genes, which are highly regulated, and all play important roles in the immune system. The Phagosome, Fc gamma R-mediated phagocytosis, Hematopoietic cell lineage, Platelet activation, and Osteoclast differentiation are important pathways that are mainly involved in the immune response. It was also found that some differentially expressed microglial genes were involved in the proliferation and regulation of multiple signaling pathways. Understanding the transcriptomic changes in microglial genes following depletion has profound implications for the activation and renewal of microglia, which may play a pivotal role in understanding the regulatory mechanism of microglial activation in diseased conditions.

## 2. Results

### 2.1. Repopulation of Microglia After Depletion

**Figure 1:**
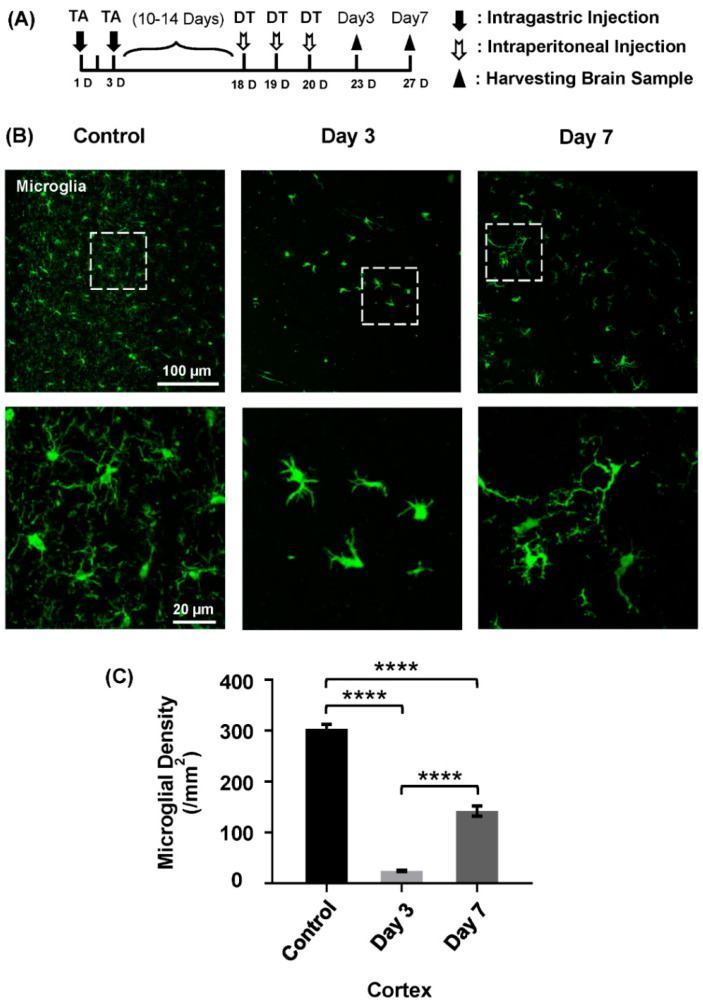
The density of cortical microglia at different time points after depletion (*n* = 3/group). (**A**) The diagram for the establishment of the experimental period of the concerned animals. (**B**) The density of the microglia in the brain’s cortex after the treatment of drugs (Tamoxifen and DT) at different time points (control, day 3, and day 7). The images in the lower panel are the magnified views of the white box regions in the upper panel. (**C**) Following microglial depletion, data showing the densities of microglia in control, day 3, and day 7 groups, and a highly significant difference in the cortex area of the brain are observed at these time points compared to the control group. Data show mean ± S.E.M.; **** *p* < 0.0001, *n* = 3/group.

To investigate the efficiency of microglial depletion in the brain, we used a microglial depletion model specifically to deplete microglia from the brain by systemic administration of DT (Figure 1A). Microglial densities were assessed at day 3 and day 7 after depletion [1]. We found that microglial densities were significantly decreased at day 3 as well as at day 7 after depletion compared to the control group (Figure 1B,C, control: 303.80 ± 5.00/mm^2^, day 3: 24.33 ± 0.95/mm^2^, and day 7: 142.20 ± 5.79/mm^2^). These results were consistent with our previous work that microglia can be successfully depleted in the brain using TA and DT administration [36].

**Figure 2:**
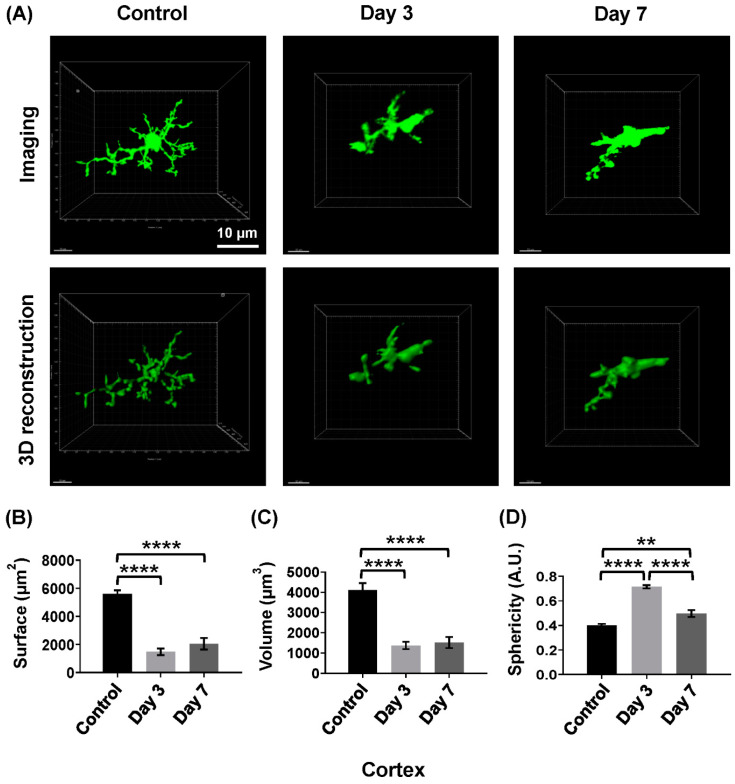
Effect of DT on the microglia of CX3CR1^CreER/+^:R26^iDTR/+^ mice. (**A**) Morphological changes of microglia in control, day 3, and day 7 groups. (**B**–**D**) Quantification of microglial morphological alterations in CX3CR1^CreER/+^:R26^iDTR/+^ mice following DT exposure. Data are shown as mean ± S.E.M.; ** *p* < 0.01, **** *p* < 0.0001, *n* = 3/group.

Notably, microglia underwent a series of transformations, with their morphology changing from a ramified form to an amoeboid form in different groups following depletion (Figure 2A). In the control group, microglia maintained their typical ramified structure, but the microglial morphology showed notable alterations from control to day 3 and day 7 groups. Quantification of morphological parameters confirmed these changes, with microglia showing a marked reduction in the surface area and the volume in day 3 and day 7 groups compared to the control group (Figure 2B,C, day 3: area: 1473.75 ± 230.80/μm^2^; volume: 1370.32 ± 179.60/μm^3^; day 7: area: 2048.36 ± 407.80/μm^2^; volume: 1521.86 ± 273.50/μm^3^; control: area: 5601.11 ± 247.60/μm^2^; volume: 4096.82 ± 354.60/μm^3^). In both the day 3 and day 7 groups, microglia exhibited a greater sphericity compared to the control group (Figure 2D), but day 7 group had a significantly reduced sphericity compared to the day 3 group (sphericity: control: 0.39 ± 0.01; day 3: 0.71 ±0.01; day 7: 0.49 ± 0.02), suggesting a progressive alteration in microglial morphology between ramified form and amoeboid form after depletion.

**Figure 3:**
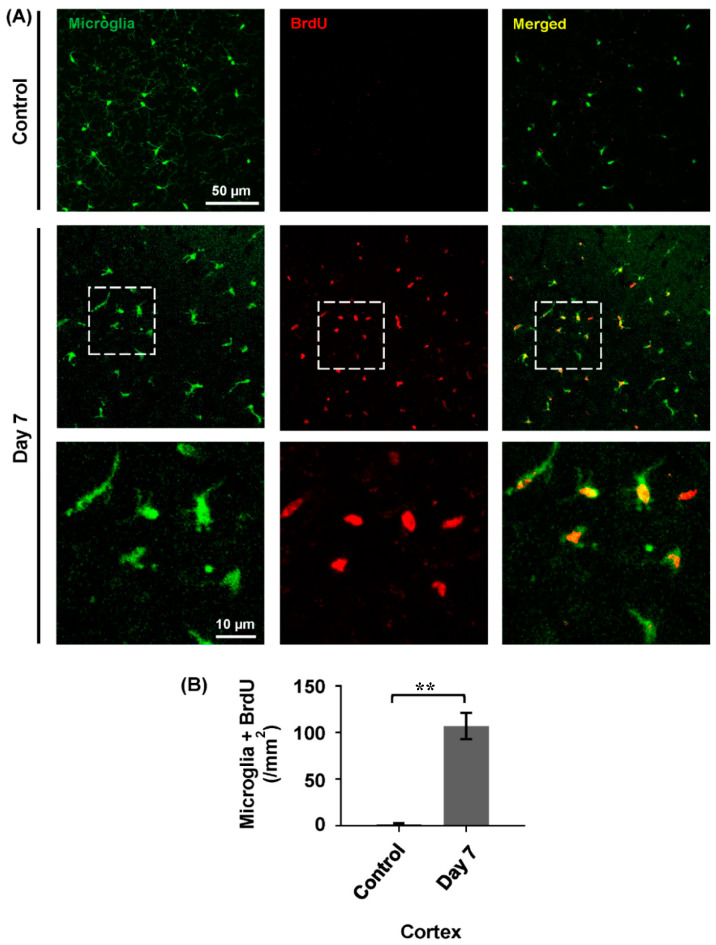
Proliferation of cortical microglia at day 7 after depletion (*n* = 3). (**A**) Confocal images showing microglia (green) and BrdU-labeled proliferating cells (red) in the cortical region of the mouse brain from the day 7 group. (**B**) Group data showing the densities of proliferative microglia in control group and proliferative microglia in day 7 groups after depletion, and significant BrdU-positive proliferative cells observed in day 7 group. Data are shown as mean ± S.E.M.; BrdU-positive proliferative cells in the control group vs. BrdU-positive proliferative cells in day 7, group ** *p* < 0.0017, *n* = 3/group.

When we compared the microglial density of the day 3 group to the day 7 group, we found there was a significant increase in microglial density. To assess whether this density increase is associated with microglial proliferation, BrdU was used to label proliferative microglia after depletion. Then, the density of BrdU-positive microglia was evaluated at day 7 after depletion (Figure 3A). The result of BrdU immunofluorescent staining showed that the density of proliferative microglia in the day 7 group was significantly higher than the control group (Figure 3B, control: 1.33 ± 0.77/mm^2^; day 7: 106.90 ± 13.96/mm^2^). These data suggest that microglia undergo active proliferation and repopulation following DT-mediated depletion in the brain.

### 2.2. Gene Expression Was Differentially Regulated at Different Time Points of Microglial Depletion

**Figure 4:**
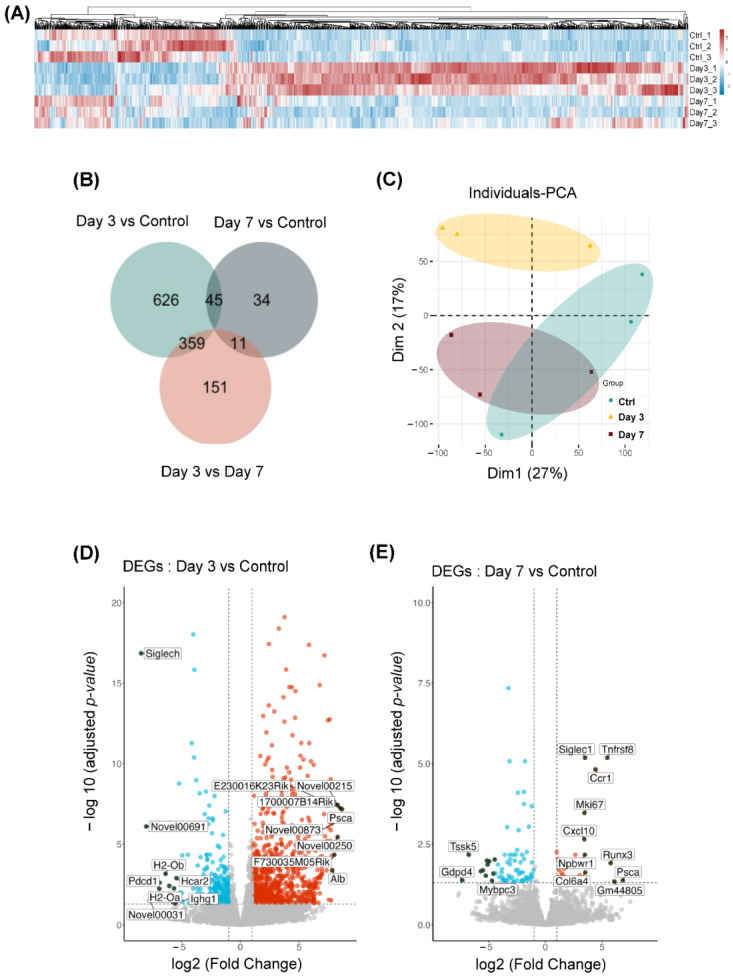
The differentially expressed genes (DEGs) after depletion of microglia. (**A**) The heat map indicates the level of DEGs (control, day 3, and day 7) after the depletion of microglia in mice (CX3CR1^CreER/+^:R26^iDTR/+^) brain by RNA-Seq. (**B**) The Venn diagram indicates the differential genes’ uniqueness to each other in comparison (*p* < 0.05) of different combinations (control, day 3, and day 7). (**C**) Principal Component Analysis (PCA) illustrating the gene expression patterns in experimental and control groups. (**D**,**E**) Volcano plot depicting the differential gene expression, with upregulated genes highlighted in red and downregulated genes in blue. The plot illustrates the number of genes that are significantly up- and downregulated.

To investigate the mechanism underlying microglial repopulation following depletion, we used data from RNA sequencing and evaluated gene expression profiles at the transcriptomic level at different time points following microglial depletion. Differentially expressed genes (DEGs) were filtered to distinguish the expression profiles in the mice of the day 3 group, day 7 group and control group. Here, the heat map shows the expression of differentially expressed genes at different time points (Figure 4A, *n* = 3/group). In total, 1030 genes in the day 3 group vs. control group, 90 genes in the day 7 group vs. control group, and 521 genes in the day 3 group vs. day 7 group were differentially expressed (Figure 4B). In these genes, a total of 767 genes were significantly upregulated, and 263 genes were downregulated in the day 3 group vs. control group. In contrast, 23 genes were upregulated and 67 genes were downregulated in the day 7 group vs. control group. When the day 3 group and day 7 group were compared, 124 genes were upregulated and 397 genes were downregulated (Figure 4D,E). We also used principal component analysis (PCA) to distinguish the gene expression patterns in all three groups. The results showed that the gene expression level on day 3 showed a significant difference to that of the day 7 group as well as the control group (Figure 4C). These results suggest that microgliosis was associated with extensive regulation of genes involved in cell proliferation at day 3 and day 7 following depletion.

### 2.3. Gene Ontology (GO) Enrichment Analysis

To explore the biological processes involved in microglial depletion and repopulation, we took advantage of the gene ontology (GO) database to identify specific GO biological processes. The GO database provides annotations that map genomic products into two aspects: the up- and downregulation GO terms in each group. We mapped the DEGs to the GO database after the depletion of microglia.

**Figure 5:**
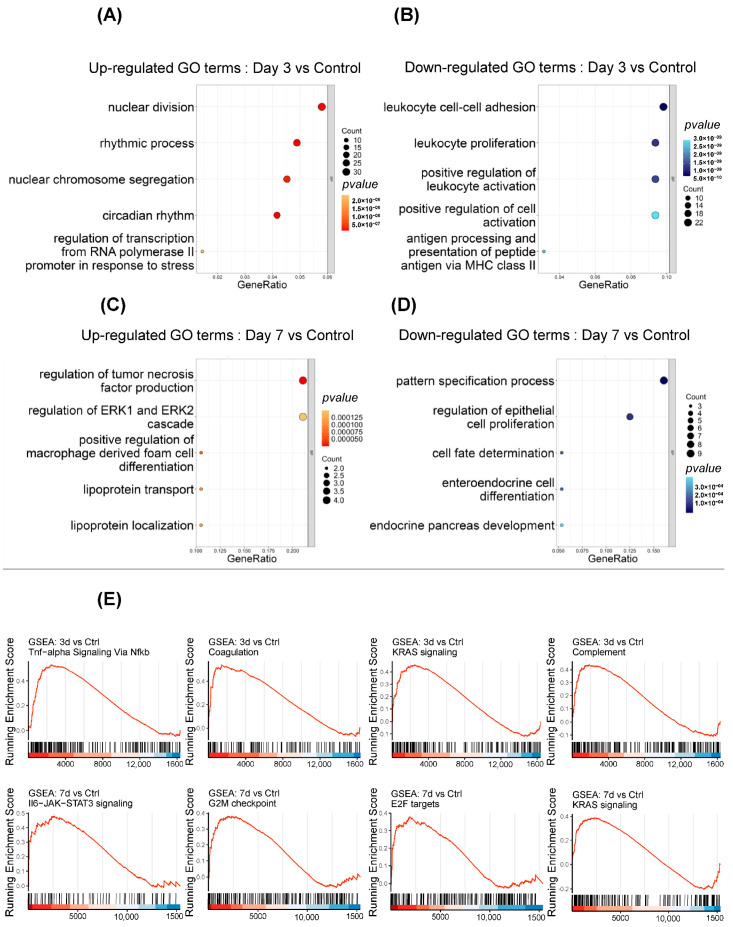
Go terms under the molecular function, cellular component, and biological processes. (**A**,**B**) Up- and downregulated Gene Ontology (GO) term enrichment of DEGs in day 3 vs. control group after microglial depletion. (**C**,**D**) Up- and downregulated Gene Ontology (GO) term enrichment of DEGs in day 7 vs. control group after microglial depletion. (**E**) Gene Set Enrichment Analysis (GSEA) shows the upregulated DEGs in day 3 vs. control and day 7 vs. control groups.

Our GO analysis results revealed that GO terms associated with nuclear division, rhythmic processes, nuclear chromosome segregation, and regulation of transcription from RNA Polymerase promoter in response to stress were enriched in day 3 vs. control (Figure 5A), highlighting changes in cellular proliferation and stress response following microglial depletion. Additionally, leukocyte-related GO terms such as leukocyte cell–cell adhesion, and leukocyte proliferation exhibited substantial enrichment, suggesting intricate regulatory mechanisms governing immune cell function. Particularly, positive regulation of leukocyte activation and positive regulation of cell activation emerged as key regulatory processes, highlighting the importance of signaling pathways modulating immune responses (Figure 5B). Moreover, on day 7 vs. control, our GO analysis unveiled regulatory mechanisms involved in the regulation of tumor necrosis factor production, regulation of ERK1 and ERK2 cascade, pattern specification process, and regulation of epithelial cell proliferation (Figure 5C,D).

### 2.4. Gene Set Enrichment Analysis (GSEA)

To analyze gene expression data in terms of predefined sets of genes that are involved in specific biological pathways or processes, we used gene set enrichment analysis (GSEA) in each group after microglial depletion. We found some key pathways/processes that play important roles in the process of activation, immune response, proliferation, and regulation [37,38,39,40,41,42,43,44]. Our results show that some pathways/processes, i.e., TNF-Alpha Signaling via NF-kB, coagulation, KRAS signaling, and complement pathways were significantly enriched after the depletion of microglia in the day 3 group vs. the control group (Figure 5E). Our results also showed remarkable enrichment of IL6-JAK-STAT3 Signaling, G2M Checkpoint, E2F targets, and KRAS signaling in the day 7 group vs. control group (Figure 5E).

### 2.5. Functional Annotations of Differentially Expressed Microglial Genes After Microglial Depletion

**Figure 6:**
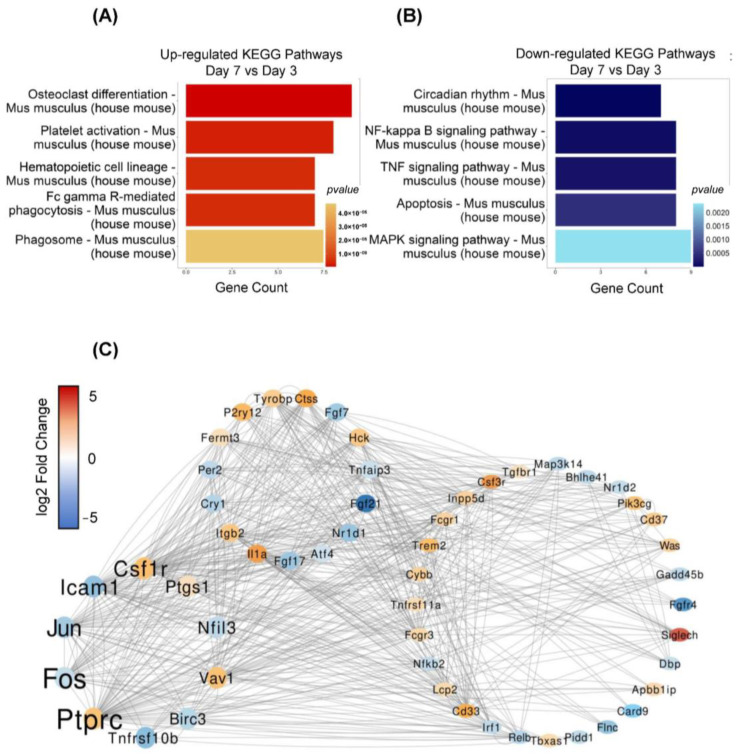
KEGG enrichment and Protein–Protein Interaction (PPI) of DEGs between day 3 and day 7 groups. (**A**,**B**) The highly up- and downregulated KEGG pathways in day 7 vs. day 3 groups. (**C**) PPI network prediction of DEGs (day 7 vs. day 3 groups) shows the key regulatory genes. Dot colors indicate the up- and downregulation of genes, while font sizes indicate the importance of the genes in the network.

To associate DEGs with specific functional pathways, we used the KEGG database to perform functional enrichment and trace genes’ function by mapping them to signaling pathway annotations. Results show that a specific KEGG term was enriched by DEGs in day 3 vs. day 7 groups. In these results, we found that some important pathways, such as osteoclast differentiation, platelet activation, hematopoietic cell lineage, Fc gamma R-mediated phagocytosis, and phagosome, were upregulated (Figure 6A), while the circadian rhythm, NF-kappa B signaling pathway, TNF signaling pathway, apoptosis, and MAPK signaling pathways were downregulated (Figure 6B).

### 2.6. Protein–Protein Interaction (PPI) Network

We used the protein–protein interaction analysis to predict the potential regulatory relationship between the DEGs and calculated the betweenness centrality of each gene. Our results showed that the genes including Ptprc, Fos, Jun, Icam1, and Csf1r were key regulators within the network. Those genes play critical roles in immune cell activation, cell immigration, and proliferation. The results indicated that the depletion of microglia promotes the differential expression of the genes related to microglial activation and proliferation. Meanwhile, Csf1r and Ptprc expression level was upregulated at day 7 compared to day 3, while Fos, Jun, and Icam1 showed downregulation (Figure 6C). These findings suggest that microglial proliferation is coordinated by the regulatory network involving the upregulation of Csf1r and Ptprc and the downregulation of Fos, Jun, and Icam1.

### 2.7. Quantitative RT-PCR Validation for Digital Gene Expression (DGE)

**Figure 7:**
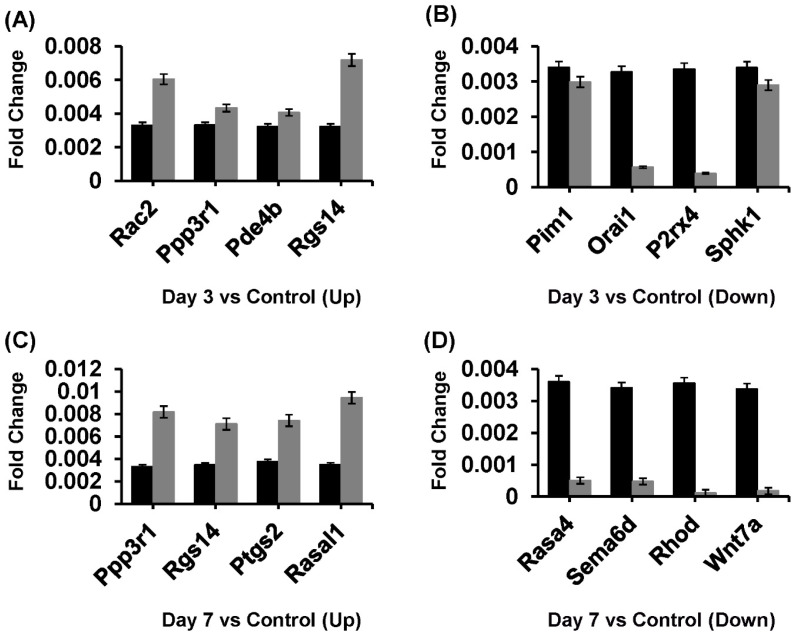
Differential regulation of microglial genes after the depletion. (**A**,**B**) The qRT-PCR fold change for four “up- and downregulated” genes was matched with log_2_ fold change DGE data on the day 3 group in response to the process of proliferation after the depletion of microglia. (**C**,**D**) The qRT-PCR fold change for four “up- and downregulated” genes was matched with log_2_ fold change DGE data on the day 7 group in response to the process of proliferation after the depletion of microglia. Here, the black columns show control, while the grey columns show day 3 and day 7 groups.

We examined the expression levels of a series of genes associated with microglial proliferation after depletion and the DEG data were then validated using qRT-PCR. Using qRT-PCR, we found that the genes Rac2, Ppp3r1, Pde4b, Rgs14 were upregulated (Figure 7A) and the genes Pim1, Orai1, P2rx4, Sphk1 were downregulated (Figure 7B) in the day 3 group vs. control group. Also, the genes Ppp3r1, Rgs14, Ptgs2, Rasal1 were upregulated (Figure 7C) and the genes, Rasa4, Sema6d, Rhod, Wnt7a were downregulated (Figure 7D) in the day 3 vs. control groups. These results are highly correlated with the transcriptome data, and the expression validation by quantitative real-time PCR (qRT-PCR) is consistent with the DGE results.

### 2.8. Differential Gene Expression Analysis of Microglial-Related Proliferation Genes, Microglial-Related Activation Genes, and Microglial-Related Chemokines

**Figure 8:**
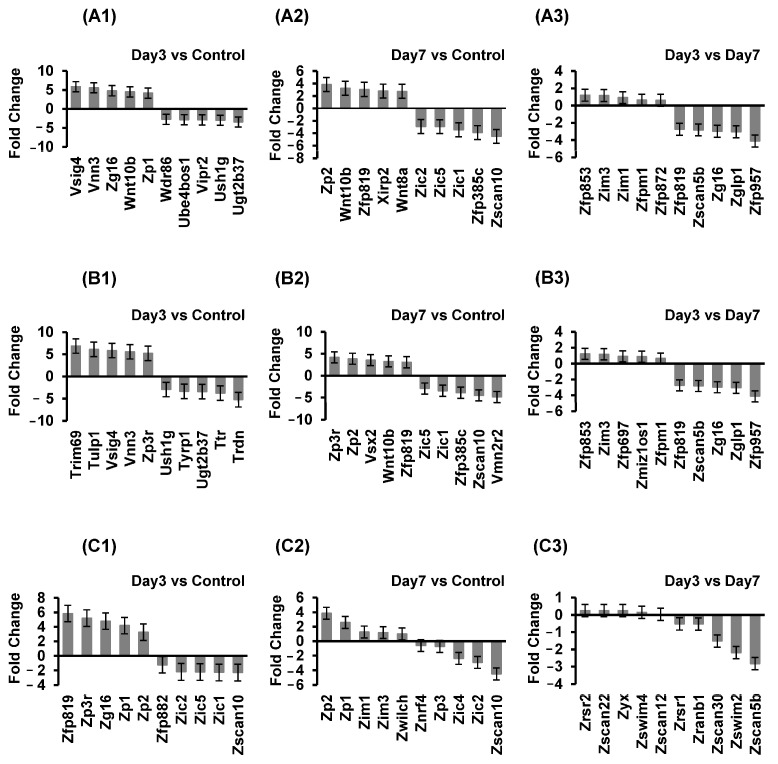
The differentially expressed microglial proliferation-related genes, microglial activation-related genes, and microglial-related chemokines after depletion. (**A1**–**A3**) The top “up- and downregulated” genes in response to the proliferation of DEG_KEGG Pathways in the day 3 group vs. control group, day 7 group vs. control group and day 3 group vs. day 7 group. (**B1**–**B3**) The top “up- and downregulated” genes in response to the activation of DEG_KEGG Pathways in the day 3 group vs. control group, day 7 group vs. control group and day 3 group vs. day 7 group. (**C1**–**C3**) The top “up- and downregulated” genes in response to the chemokines of DEG_KEGG Pathways in day 3 group vs. control group, day 7 group vs. control group and day 3 group vs. Day 7 group.

To elucidate the changes in gene expression related to microglial proliferation, activation, and related chemokines, we identified the top up- and downregulated genes at two time points (day 3 and day 7 groups) by DGE compared to the control group, as well as between day 3 and day 7 groups. The results indicate notable changes in gene expression related to microglial proliferation, highlighting potential regulatory mechanisms and pathways involved in microglial activation and response over time. Specifically, microglial proliferation-related genes V sig4, Zp2, and Zfp853 were upregulated, but Ugt2b37, Zscan10, and Zfp957 were downregulated (Figure 8A1–A3). Moreover, our findings reveal remarkable alterations in gene expression related to microglial activation at different time points, offering insights into the temporal regulation of microglial response mechanisms. Notably, Trim69, Zp3r, and Zfp853 were upregulated but Trdn, Vmn2r2, and Zfp957 were downregulated (Figure 8B1–B3). Additionally, microglial-related chemokines Zfp819, Zp2, and Zrsr2 were upregulated, but genes like Zscan10 and Zscan5b were downregulated (Figure 8C1–C3). These results shed light on the regulation of microglial chemotactic responses in different stages of activation.

### 2.9. Signaling Pathway Activation After Microglial Depletion

To associate the differentially expressed genes with specific pathways, we utilized the KEGG database to perform functional enrichment and trace gene functions by mapping them to the annotation of the signaling pathways. Results show that focal adhesion in day 3 vs. control emerged as a substantial contributor, indicating its pivotal role in orchestrating cellular interactions with the extracellular matrix (Figure S1A). Furthermore, the prominence of the VEGF signaling pathway in day 3 vs. control underscores its indispensable function in governing processes such as angiogenesis and vascular permeability modulation ( Figure S1B). The robust enrichment of the T cell receptor signaling pathway in day 7 vs. control suggests a sophisticated engagement of immune responses within our studied phenomena (Figure S1C). Increased MAPK pathway activity on day 7 highlights its crucial role in regulating cellular processes like proliferation, differentiation, and survival (Figure S1D). Collectively, these findings highlight the involvement of key pathways related to proliferation after depletion.

## 3. Discussion

In the CNS, microglia are the principal resident immune cells and play a key role in the pathological processes [10,45]. Depletion and subsequent repopulation of microglia represent intriguing avenues for understanding neuroinflammatory processes and exploring therapeutic interventions in various neurological disorders. There are different microglial depletion methods, including genetic approaches, pharmacological agents, and irradiation. These methods have been instrumental in elucidating the contributions of microglia to disease pathogenesis and in identifying potential targets for therapeutic modulations and have provided valuable insights into the functional roles of microglia in the CNS [4,46,47]. Here, we used CX3CR1^CreER/+^:R26^iDTR/+^ mice for selective microglial depletion in the brain. This genetic approach enables specific manipulation of microglia with a tamoxifen-activated CreER system and ensures precise temporal control over gene expression for accurate microglia depletion [48,49]. These advantages make CX3CR1^CreER/+^:R26^iDTR/+^ mice a powerful tool for neuroscience research, enabling detailed exploration of microglial functions and their implications in health and disease.

The repopulation process of microglia involves significant proliferation and density changes, ensuring the restoration of their population after depletion. Microglia are unique immune cells that can maintain their population by a self-renewal process. In a healthy adult brain, the density of the microglial remains relatively stable, and a reduction in microglial density is usually coupled with the proliferation of microglia [30,50]. During pathological conditions, microglia can increase their proliferative activity and ensure tissue homeostasis [30,51,52,53,54]. Here, we found that proliferating (BrdU-positive) microglia were also significantly increased after depletion. Similar repopulation dynamics were reported in the microglial depletion model that used acute diphtheria toxin [31], which also confirmed that microglial repopulation occurred within the CNS.

DGE was used for the transcriptomic analysis of microglial depleted tissue in mouse brain. Our DGE data revealed the expression of some key microglial genes associated with proliferation following microglial depletion. Specifically, we found that Pim1, Rac2, P2rx4, and Ptgs2 are regulated in response to microglial proliferation. Previous research indicates that Pim1 and Rac2 play crucial roles in microglial proliferation, mobility, and migration, which are essential for effective immune responses [55,56]. Additionally, P2rx4 and Ptgs2 are involved in cellular activation and proliferation, contributing to the pro-inflammatory signaling pathways in microglia [57,58]. Together, these genes seem to work to regulate microglial proliferation and migration after depletion. All these data suggest that microglia may uniquely and efficiently restore their population and maintain homeostasis in the brain.

One of the unique features is that microglia can undergo morphological changes after activation, as revealed by the de-ramification of their distinctly dynamic processes. Upon activation, microglia transform from a ramified state with long, branched processes into an amoeboid form, characterized by shorter and thicker processes [30,59], and after depletion, we observed that on day 7, microglial morphology transformed from de-ramified to ramified form, suggesting that microglia transformed from an activation-like state to a resting-like state after depletion.

To investigate key molecules involved in triggering or regulating microglial activation, we used PPI network analysis to evaluate the potential regulatory relationship between the DEGs and calculate the betweenness centrality of genes. We found that Ptprc, Fos, and Csf1r were key regulators within the network. Studies found that Csf1r is a critical factor that controls microglial proliferation in both the healthy and injured brain [4,60,61]. Csf1r is necessary for microglial proliferation and viability and Csf1r is involved in triggering or regulating the activation, migration, and differentiation of microglia [4,62,63,64,65,66]. Our findings show that Csf1r plays a critical role in the differential regulation of microglial proliferation following depletion.

Another important regulator of microglial activity is Ptprc. Ptprc, also known as CD45, is a critical regulator of microglial proliferation. Here, we found that Ptprc showed high connectivity in the network and plays a crucial role in brain homeostasis and neuroinflammatory conditions. Studies show that Ptprc plays a significant role in modulating microglial activation and inflammatory responses, which are essential for maintaining brain homeostasis [67,68]. Moreover, the absence or malfunction of Ptprc has been linked to aberrant microglial proliferation, indicating its importance in neuroinflammatory conditions [69]. These findings indicate that Ptprc is essential for regulating microglial activity and proliferation.

Fos is another key molecule that is involved in the regulation of microglial activity. Studies show that Fos has been associated with proliferation, differentiation, transformation, and cellular death [70,71,72,73]. In PPI network analysis, we found that Fos represented the higher degree of association in the network after microglial depletion. These findings underscore the potential of Fos as a therapeutic target in conditions characterized by microglial proliferation.

Our data revealed a large number of cellular processes (nuclear division, rhythmic processes, nuclear chromosome segregation, and transcription regulation from RNA Polymerase II promoter in response to stress) and signaling pathways (focal adhesion signaling pathway, VEGF (vascular endothelial-derived growth factor) signaling pathway, T cell receptor (TCR) signaling pathway, and MAPK (mitogen-activated protein kinase) signaling pathway) responded to microglial depletion. Studies showed that these cellular processes and signaling pathways are actively involved in the process of proliferation [74,75,76,77,78,79,80,81]. Our findings indicate that these cellular processes and signaling pathways are highly regulated and deeply involved in microglial proliferation following depletion, suggesting a pivotal role of these cellular processes and signaling pathways in microglial proliferation after depletion.

## 4. Materials and Methods (Experimental Procedure)

### 4.1. Animals

In this study, mice (CX3CR1^CreER/+^:R26^iDTR/+^) of both sexes with a C57/BL6 background were selected as experimental subjects. All transgenic mice (weight 18–25 g, 2–4 months old) were bred in-house under standard environmental and nutritional conditions throughout the study (temperature 22 ± 2 °C, humidity 55 ± 10%, light-dark cycle 12:12, with food and water available ad libitum). All animal experiments were performed following ethical approval by the Ethics Committee of Lanzhou University, China.

### 4.2. Microglia Depletion Model

Tamoxifen (Sigma-Aldrich, St. Louis, MO, USA, Cat# T5648) dissolved in corn oil was administered to adult mice by gavage. All the animals received two intragastric injections of tamoxifen doses at 48 h intervals. Diphtheria toxin (DT) (Sigma-Aldrich, Cat# D0564) was diluted in DPBS (Dulbecco’s phosphate buffered saline), and 1 μg of diphtheria toxin was given by intraperitoneal (IP) injection for three consecutive days to deplete the microglia. Our preliminary data indicated that a remarkable repopulation of microglia occurred during the first week after depletion. Therefore, we have chosen day 3 and day 7 as the representative time points to analyze the differential expressions of microglial genes during repopulation. Animals were divided into 3 groups: the control group, the day 3 group, and the day 7 group.

### 4.3. Tissue Preparation

Mice were anesthetized with 15% ethyl urethane and then transcardially perfused with 0.01 M phosphate-buffered saline (PBS). The brains were immediately removed and placed into liquid nitrogen for two minutes to freeze. Total brain tissue was used to extract the RNA using the TRIzol^®^ reagent (Invitrogen, Carlsbad, CA, USA) for RNA sequencing. RNA contamination and degradation were assessed using a 1% agarose gel, and purity was checked using a Nano Photometer^®^ and spectrophotometer (IMPLEN, Westlake Village, CA, USA). RNA concentration was measured using the Qubit^®^ RNA Assay Kit in a Qubit^®^ 2.0 Fluorometer (Life Technologies: Carlsbad, CA, USA). Finally, RNA purity was verified using the RNA Nano 6000 Assay Kit on the Agilent Bioanalyzer 2100 system (Life Technologies, CA, USA).

### 4.4. Immunohistochemistry and Histology

For histological and immunohistochemical analysis, all mice were anesthetized with urethane and then perfused transcardially with 0.1 M phosphate-buffered saline (PBS) followed by 4% paraformaldehyde (PFA). The brains of all samples were removed and postfixed in 4% PFA at 4 °C for 24 h. Subsequently, each brain was sectioned into 30 µm thick coronal sections using a Leica vibrating microtome. The first antibody (Rabbit antibody) specific for ionized calcium-binding adaptor molecule-1 (IBA-1; 1:500) was used (IBA1; Wako Pure Chemical Industries, Osaka, Japan) and incubated at 4 °C overnight to stain for IBA-1. Finally, the brains were collected and sectioned into coronal slices for analysis at day 3, day 7, and in the control group after microglia depletion.

To label newly proliferative cells during microglial depletion, bromodeoxyuridine (BrdU, 50 mg/kg; Sigma-Aldrich, St. Louis, MO, USA) was intraperitoneally injected into the mice two times per day, and the mice brain tissues were extracted, fixed with 4% paraformaldehyde solution and sectioned. BrdU immunofluorescence staining was conducted to label proliferating cells following depletion, as previously described [53]. The brain sections (30 μm) were rinsed in PBS for 10 min, treated with 2M HCl at 37 °C for 30 min, neutralized with 0.1 M borate buffer (pH 8.5) for 3 × 15 min, and then incubated overnight with a rat monoclonal anti-BrdU antibody (1:500; AbD Serotec, Hercules, CA, USA). After three washes, the sections were stained with rhodamine-conjugated goat anti-rat secondary antibody (1:100; ZSGB-BIO, Beijing, China) for 1 h at room temperature. The samples were sealed with a PBS/glycerol mixture (2:3) and examined using a confocal microscope. Microglia (green) co-localizing with BrdU-positive cells (red) were identified as proliferative microglia.

### 4.5. Confocal Microscopy

To photograph the fluorescently stained tissue samples, a laser scanning microscope (Olympus Flue-View FV1000, Olympus Corporation: Tokyo, Japan) was utilized. The confocal photographs were captured into a 60 μm z-stack at 1 μm intervals with a 40×/0.95 NA objective. Three slices were selected from each brain sample, and the microglia density in each slice of the mouse’s brain was assessed using ImageJ software “http://rsb.info.nih.gov/ij/ (accessed on 1 September 2024)”.

### 4.6. Quantitative Real-Time PCR (qRT-PCR) Analysis

Total mRNA was extracted from tissue samples using the Bio-Teke Kit (RP1202, Bioteke Corporation: Beijing, China) following the manufacturer’s protocol. For quantitative real-time PCR, cDNA was synthesized using a SYBR Premix Ex Tag II (RR820A, TaKaRa Corporation: Shiga, Japan) and random hexamer primers according to the manufacturer’s protocols. qRT-PCR analysis was performed using a CFX96 TM Real-Time PCR Detection System (Bio-Rad Laboratories, Inc., Hercules, CA, USA) with a SYBR Premix Ex Tag TM II (RR820A, TaKaRa Corporation). The samples underwent 40 cycles of amplification at 95 °C for 30 s, 60 °C for 1 min, 50 °C for 2 min, and 95 °C for 10 min. GAPDH (glyceraldehyde-3-phosphate dehydrogenase) was used as a reference gene. Triplicates of each reaction were taken out, and the targeted gene expression level was normalized to GAPDH as an internal reference. The mRNA level of each target gene was expressed as 2^∆Ct^ (∆Ct = Ct target−Ct GADPH). The relative quantity of mRNA levels of tested genes was determined using the following equation: relative quantity = 1000/2^∆Ct^. The custom-designed gene-specific primers (GENEWIZ, Inc., South Plainfield, NJ, USA) are shown in Supplementary Table S1.

### 4.7. RNA Sequencing and Library Construction

RNA sequencing was conducted on mRNA obtained from the control, day 3, and day 7 groups following microglial depletion. Libraries were generated using the NEBNext^®^ UltraTM RNA Library Prep Kit for Illumina (NEB, Ipswich, MA, USA), and sequencing was performed following the manufacturer’s recommendations. Magnetic poly-T oligo beads were utilized to isolate the mRNA from 3 μg of RNA. The mRNA fragmentation was performed in the NEBNext First-Strand Synthesis Reaction Buffer (5×) at elevated temperatures. The first- and second-strand cDNA was synthesized using M-MuLV Reverse Transcriptase (RNase H), DNA polymerase I, and RNase H with a random hexamer primer. Exonuclease/polymerase was used to convert the remaining overhangs into blunt ends. The adenylation of 3′ ends of DNA fragments was followed by ligation with a NEBNext Adaptor and a hairpin loop structure for hybridization. The cDNA fragment of 150–200 bp was obtained from the library fragments using the AMPure XP system (Beckman Coulter, Inc., Beverly, MA, USA). The size-selected adaptor-ligated cDNA was treated with 3 μL USER Enzyme (NEB, USA) at 37 °C for 15 min, followed by 5 min at 95 °C before PCR amplification. The PCR was performed using Phusion High-Fidelity DNA polymerase, Universal PCR primers, and an Index (X) Primer. The PCR products were purified using the AMPure XP system, and the quality of the library was assessed based on the Agilent Bioanalyzer 2100 system.

### 4.8. Quantification of Transcripts by RNA-Seq

The above data of RNA sequencing indicate a great number of differentially expressed genes (DEGs). After filtering, only microglial genes were used for the analysis with the Illumina platform (Agilent 2100). Clean reads were obtained by removing the reads containing the adapter, poly N, and other low-quality reads. And the high-quality reads were then aligned to the reference genome of the mouse, yielding a high mapping rate. The significant characteristics of the day 3 group, day 7 group post-microglial depletion, and tag libraries are summarized in Table S2. To assess the reproducibility of the differential gene expression (DGE) among the day 3 group, day 7 group, and control group, we applied Pearson’s correlation analysis. The results showed high correlation and reproducibility among individual samples within each group. We leveraged previously conducted tag studies and databases to associate gene expression with specific cell types in the CNS. Individual genes were analyzed by using two databases: Mouse Genome Informatics (MGI) “www.informatics.jax.org/genes.shtml (accessed on 15 March 2019)” and Brain RNA Sequencing “http://web.stanford.edu/group/barres_lab/brain_rnaseq.html (accessed on 15 March 2019)” [82]. To develop the box plot of the FPKM (Fragments Per Kilobase of exon per Million reads) values, RNA sequencing was used to analyze differentially expressed genes among different groups. The threshold for identifying differentially expressed genes was set at a corrected *p*-value (*p*-value) < 0.005 and a log2 fold change > 1.

### 4.9. The Read Mapping and the Quantification of the Gene Expression Level

Raw reads in Fastq format were prepared using in-house Perl scripts. Clean reads were obtained from the raw data by removing reads containing low-quality sequences, poly-N regions, and adapters. The clean reads were then analyzed to calculate GC content, Q20 content, and Q30 content. Reference gene and genome model annotation files were retrieved from a genomics website. HT-Seq (V0.61) was utilized to count the mapped read numbers for each gene. Quantification was performed by assembling RNA-Seq reads into transcripts, and their abundance was estimated using the FPKM method [83]. To align paired-end clean reads, TopHat (V2.0.12) was employed to build an index of the reference genome, and Bowtie (V2.2.3) was used for the alignment process.

### 4.10. The DGE Analysis

The differentially expressed genes of the three groups were analyzed using the DESeq2 [84] package under the R environment (version 4.3.3). Using Benjamini and Hochberg’s approach, the resulting *p*-value was adjusted. To control the false analysis rate, the *p*-value was adjusted by using the corrected *p*-value (*p* < 0.005), and log2 (fold change) > 1 was used as a threshold for the significant differential analysis results.

### 4.11. The KEGG and GO Enrichment Analysis

The over-enrichment analysis of DEGs was performed with the package ‘clusterProfiler’ under the R environment. The pathway analysis is primarily based on the Kyoto Encyclopedia of the Genes and the Genomes (KEGG) database [85]. The signal transduction pathways or the significantly enriched pathways were identified using KEGG’s database in the differentially expressed genes. The hypergeometric test was used to map all differentially expressed genes (DEGs) to terms in the Gene Ontology (GO) database. This mapping was performed in conjunction with the Kyoto Encyclopedia of Genes and Genomes (KEGG) pathway database. The purpose of this mapping was for gene expression profile analysis. Additionally, it was used for GO enrichment analysis of functional significance. The False Discovery Rate (FDR) < 0.05 values indicate a significant enrichment of differentially expressed genes in a particular pathway. To test the statistical enrichment of differential gene expressions and analyze the enrichment of pathways in the Kyoto Encyclopedia of Genes and Genomes (KEGG), the KOBAS (2.0) software was utilized.

### 4.12. Statistical Data Analysis

The cell density was quantified by randomly selecting three rectangular regions, each measuring 300 μm × 300 μm, from cortex. Statistical analysis was conducted using Graph Pad Prism Version 6.02 software (La Jolla, CA, USA). An unpaired *t*-test (two-tailed) was employed between two different groups and a one-way analysis of variance (ANOVA) was used for the comparison of more than three groups. All the data are presented as the mean ± S.E.M. Significance levels are denoted as * *p* < 0.05, ** *p* < 0.01, *** *p* < 0.001, and **** *p* < 0.0001.

## 5. Conclusions

In conclusion, our RNA-sequencing analysis dataset not only characterizes the dynamic changes in gene expression but also provides a list of differentially expressed genes and regulated pathways involved in microglial depletion. DEG analysis revealed many differentially expressed genes at different days following microglial depletion, predominantly those specific to microglia. These genes play a key role in various gene ontology (GO) categories, including proliferation, activation, and chemokine production. Our findings suggest that the observed transcriptomic changes after microglial depletion have profound implications for the activation and renewal of microglia. Furthermore, these results may contribute to a better understanding of the regulatory mechanisms underlying microglial activation in diseased conditions.

## Data Availability

The data supporting the conclusions of this article are available from the authors on request.

## Supplementary Materials

The following supporting information can be downloaded at: https://www.mdpi.com/article/10.3390/ijms26041494/s1.
