# Supplementary material for: Transcriptomic Analysis Divulges Differential Expressions of Microglial Genes After Microglial Repopulation in Mice
Source: Int J Mol Sci. 2025 Feb 11;26(4):1494. doi: 10.3390/ijms26041494 (PMC11855859; doi:10.3390/ijms26041494)
Supplement: Supplementary file 1 [file ijms-26-01494-s001.zip › ijms-3395237-supplementary.pdf]

Primers	5' – 3' forward primers	5' – 3' reverse primers
**Rac2**	TTGACAACTACTCAGCCAATG	CGTACCTCAGGGAACCAC
**Ppp3r1**	GCCCTGTTCCGTGCCTT	CGGTTGGAGATGCGTCGG
**Pde4b**	TCGGAATCTGAGGTTTG	GTATGAGCCACCGTGAA
**Rgs14**	CAAGGACAGTCACCTTCCCC	AGGCAGGGCACCTTCAATG
**Pim1**	GCGGCGAAATCAAACTC	TCGTGCTCAAACGGAATAT
**Orai1**	CATCGGGACGCTGCTTT	GCTGCTGTCGCTGTGGTT
**P2rx4**	TGGAGAATGACGCTGGTG	GACTTGAGGTAGGAGGTGGT
**Sphk1**	GTTTGTAGTAGAATGCCCTCG	TTCTTCCGTTCGGTGAGTA
**Rgs14**	CAAGGACAGTCACCTTCCCC	AGGCAGGGCACCTTCAATG
**Ptgs2**	TCAGGTCATTGGTGGAG	CACTCTGTTGTGCTCCC
**Rasal1**	GCTGCTGACCGGATACCT	AGAACCGCAGAAAGAGGAA
**Rasa4**	CACCAGTGAAGCCAACACC	CCAGAGCACCCTACATCCTT
**Sema6d**	TGCCCTTCGCACAATAA	GCCACCCATGTCGTTTT
**Rhod**	TCAAGGTGGTCCTGGTGG	CGGAGGCGGTCATAGTCA
**Wnt7a**	CTGGACGAGTGTCAGTTTCA	CACAGTCGCTCAGGTTGC

Supplementary Table S1. Forward and reverse primers are used for qRT-PCR. Here the gene-specific primers of microglial proliferation were shown.

Sample name	Control 1	Control 2	Control 3	Day 3-1	Day 3-2	Day 3-3	Day 7-1	Day 7-2	Day 7-3
**Total reads**	**64589290**	**63877718**	**49868716**	**56023174**	**62963660**	**60218128**	**57493874**	**61796930**	**63042060**
**Total mapped**	**58107086<br>(89.96%)**	**57344578<br>(89.77%)**	**44962686<br>(90.16%)**	**50306877<br>(89.8%)**	**56917143<br>(90.4%)**	**54349436<br>(90.25%)**	**51113897<br>(88.9%)**	**55668587<br>(90.08%)**	**56909134<br>(90.27%)**
**Multiple mapped**	**1427391<br>(2.21%)**	**1353559<br>(2.12%)**	**1058321<br>(2.12%)**	**1190159<br>(2.12%)**	**1288030<br>(2.05%)**	**1311178<br>(2.18%)**	**1191969<br>(2.07%)**	**1262409<br>(2.04%)**	**1372563<br>(2.18%)**
**Uniquely mapped**	**56679695<br>(87.75%)**	**55991019<br>(87.65%)**	**43904365<br>(88.04%)**	**49116718<br>(87.67%)**	**55629113<br>(88.35%)**	**53038258<br>(88.08%)**	**49921928<br>(86.83%)**	**54406178<br>(88.04%)**	**55536571<br>(88.09%)**
**Read-1**	**28448848<br>(44.05%)**	**28171947<br>(44.1%)**	**21987469<br>(44.09%)**	**24680633<br>(44.05%)**	**27883331<br>(44.28%)**	**26575589<br>(44.13%)**	**25094844<br>(43.65%)**	**27292183<br>(44.16%)**	**27902326<br>(44.26%)**
**Read-2**	**28230847<br>(43.71%)**	**27819072<br>(43.55%)**	**21916896<br>(43.95%)**	**24436085<br>(43.62%)**	**27745782<br>(44.07%)**	**26462669<br>(43.94%)**	**24827084<br>(43.18%)**	**27113995<br>(43.88%)**	**27634245<br>(43.83%)**
**Reads map to '+'**	**28323613<br>(43.85%)**	**27970494<br>(43.79%)**	**21925376<br>(43.97%)**	**24527299<br>(43.78%)**	**27781729<br>(44.12%)**	**26494065<br>(44%)**	**24934840<br>(43.37%)**	**27172913<br>(43.97%)**	**27732188<br>(43.99%)**
**Reads map to '-'**	**28356082<br>(43.9%)**	**28020525<br>(43.87%)**	**21978989<br>(44.07%)**	**24589419<br>(43.89%)**	**27847384<br>(44.23%)**	**26544193<br>(44.08%)**	**24987088<br>(43.46%)**	**27233265<br>(44.07%)**	**27804383<br>(44.1%)**

Supplementary Table S2. Overview of mapping status of differentially expressed genes (DEGs) reads after the depletion of microglia in mice (CX3CR1$^{CreER/+}$: R26$^{iDTR/+}$) brain by RNA-Seq.

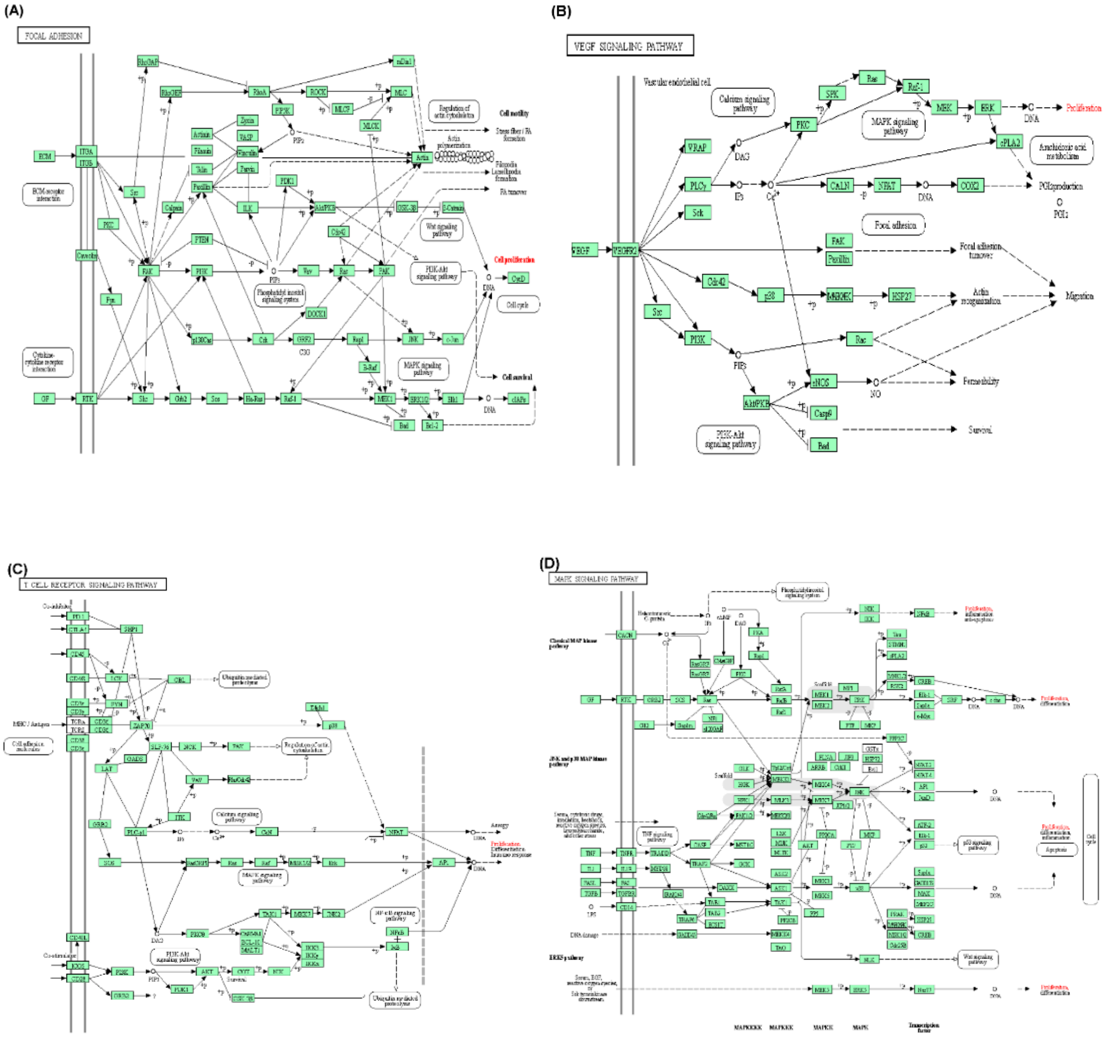

Supplementary Figure S1. KEGG analysis signaling pathways. **(A, B)** Proliferation-related signaling pathways displaying differentially expressed genes following microglia depletion at Day 3 compared to the control group. **(C, D)** Proliferation-related signaling pathways showed differentially expressed genes after microglia depletion at Day 7 compared to the control group.
